# Transferrin Receptor 2 in Canine Testicular Tumors: An Emerging Key Role in Seminomas

**DOI:** 10.3390/ani15020264

**Published:** 2025-01-18

**Authors:** Rebecca Leandri, Sara Buonocore, Karen Power

**Affiliations:** Department of Biology, University of Naples Federico II, 80126 Naples, Italy; rebecca.leandri@unina.it (R.L.); sara.bcore@gmail.com (S.B.)

**Keywords:** canine testicular tumor, immunohistochemistry, iron, TfR2, seminoma, therapy

## Abstract

Transferrin Receptor 2 (TfR2) is a homolog of Transferrin Receptor 1 (TfR1), involved in regulating intra and extracellular iron levels. Altered iron pathways have been associated with cancer onset and progression; however, their role in canine tumors remains poorly explored. This study investigated TfR2 immunohistochemical expression in non-neoplastic canine testis for the first time and in the most common types of canine testicular tumors: intratubular seminomas (ITSEMs), diffuse seminomas (DSEMs), Leydig cell tumors (LCTs), and Sertoli cell tumors (SCTs). Immunohistochemical analysis revealed a differential pattern of TfR2 expression according to tumor type, with high expression observed in ITSEMs and DSEMs, occasional expression in LCTs, and absence in SCTs. These results suggest that TfR2 may play a relevant role in canine seminoma development. Furthermore, the specific expression of TfR2 in seminomas highlights its potential as a therapeutic target, where its role in iron regulation and possible compensatory mechanisms warrant further investigation.

## Simple Summary

This study investigates the expression of Transferrin Receptor 2 (TfR2) in canine testicular tissues, focusing on its role in iron metabolism and its potential as a therapeutic target. We observed high TfR2 expression in seminomas, suggesting its involvement in tumor progression and highlighting its potential for targeted cancer therapies. By contrast, Sertoli and Leydig cell tumors exhibited little to no TfR2 expression, emphasizing distinct patterns of iron metabolism in different tumor types. These findings open avenues for further research into TfR2-based therapeutic strategies in canine testicular tumors.

## 1. Introduction

Testicular cancer is one of the most common tumors found in male dogs [1,2,3,4]. According to the histological classification of tumors of domestic animals [5], three histotypes can be identified: seminomas (SEMs) (24–42% of testicular tumors), Interstitial cell tumors (ICTs) (23–51%), and Sertoli cell tumors (SCTs) (8–33%) [6,7,8,9]. Moreover, SEMs can be further classified as intratubular (ITSEMs) or diffuse (DSEMs) [10]. While surgical removal of testicles is an effective approach for treating testicular tumors, castration could favor secondary diseases such as canine prostatic neoplasia (CPN) and other neoplastic disorders [11,12,13,14,15]. For this reason, new therapeutical approaches are needed. Since spontaneous tumors in companion animals, especially canine ones, share several pathophysiological and clinical similarities with human cancers, they can be studied to find translational therapeutic targets [16]. Canine testicular seminoma presents similar histological structures as human ones in elderly men, suggesting their relevance as potential models [17].

Iron metabolism plays a crucial role in maintaining cellular homeostasis [18,19,20]; in particular, it is involved in key processes of cell functionality such as energy metabolism and oxygen balance [21,22]. Furthermore, iron is crucial in regulating reproductive functions: (1) it is necessary to maintain testosterone synthesis [23]; (2) it is correlated to the growth, maturation, and release of germ cells and to the correct functionality of Sertoli cells [24]; (3) its excess can induce ferroptosis and affect sperm quality [25]. In physiological conditions, iron uptake in the testis occurs through TfR1, a cell-surface receptor expressed in spermatogonia, pachytene spermatocytes and Sertoli cells [26]. In these cells, TfR1 binds the iron-carrier protein Transferrin (Tf)-Fe3+ complex [27,28], and through an endocytic process known as the Tf cycle, iron is internalized in the cytosol where it is reduced to Fe^2+^ by ferrireductases [29], released from Tf and stored in the cytoplasm by Ferritin (Ft) [30]. Dysregulation of iron metabolism has been linked to tumor onset and progression [31]. Previous studies have highlighted that iron metabolism can be disrupted in different canine and human tumors [32,33,34,35,36,37,38,39,40]. In a previous study from our research group [32], we described the expression of iron-related proteins, namely Transferrin Receptor 1 (TfR1), Ferritin (FTH1) and Nuclear Co-factor (NCOA4) in non-neoplastic canine testicles and their altered expression in neoplastic testis. Expression of the aforementioned proteins is related to and depends on the tumor type; in particular, DSEMs showed a significant increase in TfR1 expression in neoplastic germ cells. Transferrin Receptor 2 (TfR2) is a homolog of TfR1 and it can also bind circulating Tf, although with lower affinity [41,42]. Their major differences concern their expression pattern: TfR1 expression is reported in various tissues, while TfR2 expression is reported mainly in the human liver [43], and, conversely to TfR1, it is not regulated by intracellular iron levels but it seems to be regulated by the cell cycle stages, at least in humans [44,45]. Moreover, previous studies reported frequent TfR2 expression in tumor cell lines, particularly in human ovarian cancer, colon cancer and glioblastoma cell lines [46]. Unfortunately, little is known about TfR2 expression in canine tumors. In this study, we provide evidence for TfR2 preferential expression in canine seminomas, suggesting TfR2 as an additional therapeutical selective target in the abovementioned tumors.

## 2. Materials and Methods

### 2.1. Tissue Samples

Twenty-nine testicular specimens were obtained from the archives of the Department of Biology at the University of Naples Federico II. For each specimen, data on age, breed and histological diagnosis were available, as summarized in Table 1.

### 2.2. Histology

The specimens were fixed in 10% formalin and subsequently processed for routine histopathological analysis as described by Leandri et al. [32]. Sections measuring 3 μm were prepared from paraffin-embedded blocks and stained with hematoxylin and eosin (H&E) for light microscopy examination. The ethics committee’s approval and animal testing request was waived since all animal tissue samples examined in this study were retrieved from archives.

### 2.3. Immunohistochemistry

Additional 3 μm sections were prepared for immunohistochemistry (IHC) to assess the expression of the studied protein TfR2 as previously described [32]. Details and dilution of the antibody here used are provided in Table 2. Immunolabelling was visualized using diaminobenzidine–tetrahydrochloride (DAB), and sections were counterstained with hematoxylin. Specimens were observed and photographed using a light microscope (AXIO SCOPE.A1, Carl Zeiss S.p.A., Oberkochen, Germany) equipped with a microphotography system digital camera (Axiocam 105 color, Carl Zeiss S.p.A., Oberkochen, Germany).

### 2.4. Western Blot

To ensure the specificity of immunolabelling, Western blotting analysis was performed for the TfR2 antibody.

A small portion of the testicular tissue specimens was used for total protein extraction and subsequently processed as previously described [32]. Table 3 shows antibody details and dilutions used for Western blot analysis. After washing with Tris-buffered saline containing 0.1% Tween-20, membranes were incubated for 1 h at room temperature with secondary antibodies: goat anti-rabbit IgG-HRP conjugate (1:5000; EMD Millipore, 12-348; Darmstadt, Germany). Chemiluminescent signals were generated by incubating the membranes with an enhanced chemiluminescence substrate (Clarity Western ECL substrate, 1705061, Bio-Rad, Hercules, CA, USA) for 5 min at room temperature. Signals were detected using the XRS+ Chemidoc Imaging System (Bio-Rad, Hercules, CA, USA). Biological duplicates were performed to ensure the reproducibility of the results.

## 3. Results

### 3.1. Non-Neoplastic Testis

**Figure 1:**
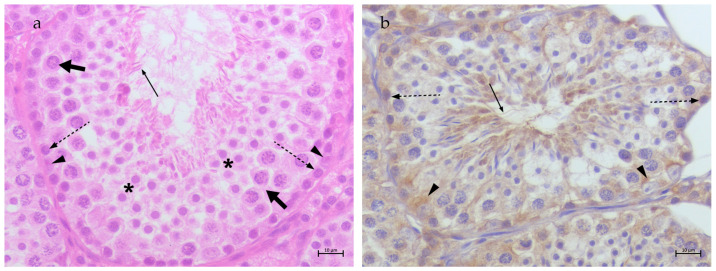
(**a**) Testis, non-neoplastic: spermatogonia (dotted arrows), spermatocytes (fat arrows), spermatids (asterisks), spermatozoa (thin arrows) and Sertoli cells (arrow heads) can be observed. H&E, 40× Bar 10 μm; (**b**) TfR2 immunostaining in canine non-neoplastic testis: low perinuclear TfR2 signal in spermatogonia (dotted arrows), cytoplasmatic low signal in Sertoli cells (arrow heads) and in spermatozoa tails (thin arrows); 40× Bar 10 μm.

Samples N1–N3 represented non-neoplastic testes and were used as controls. H&E staining (Figure 1a) confirmed complete spermatogenesis, with fully formed seminiferous tubules containing Sertoli cells and germinal cells from the stage of spermatogonia to the stage of spermatozoa. In the interstitial tissue, Leydig cells were observed. In all non-neoplastic samples, a low membranal anti-TfR2 signal was detected in spermatogonia at the perinuclear site, Sertoli cells cytoplasm and spermatozoa tails (Figure 1b).

### 3.2. Neoplastic Testis

**Figure 2:**
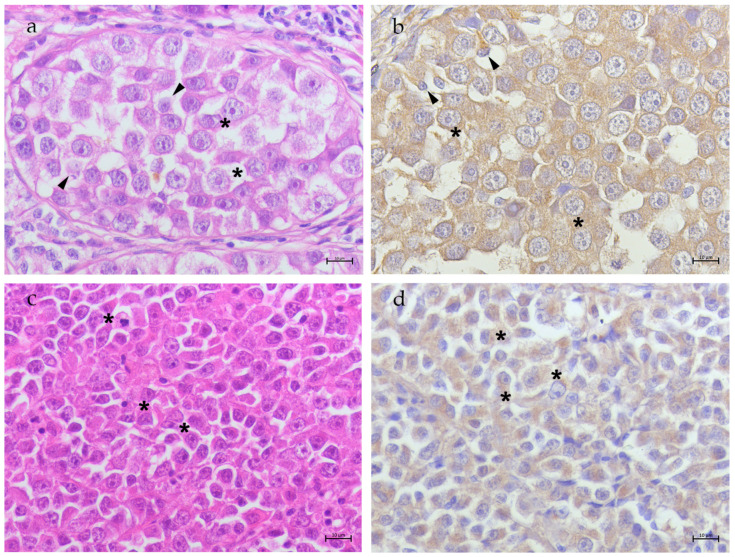
Testis, seminoma. (**a**) ITSEM: seminiferous tubule showing large neoplastic cells (asterisks) and a few Sertoli cells (arrow heads). H&E 40× bar 10 μm; (**b**) TfR2 immunostaining in ITSEM: high cytoplasmatic TfR2 immunostaining in neoplastic (asterisks) and Sertoli cells (arrow heads); 40× bar 10 μm; (**c**) DSEM: sheets of small round/oval neoplastic cells (asterisks). H&E 40× bar 10 μm; (**d**) TfR2 immunostaining in DSEM: high cytoplasmatic TfR2 immunostaining in neoplastic cells (asterisks); 40× bar 10 μm.

*Seminomas*—ITSEMs (S6–S11) displayed cells with large round nuclei, evident nucleoli, and finely granular chromatin, along with moderate amounts of pale to clear cytoplasm. Sertoli-like cells within the tubules showed round to typical flame-shaped cytoplasm and small nuclei (Figure 2a). In ITSEM samples, TfR2 was highly expressed in the cytoplasm of neoplastic cells and residual Sertoli-like cells within the tubules (Figure 2b). Conversely, DSEM (S12–S23) showed oval to round cells with abundant cytoplasm, prominent nuclei and multiple nucleoli (Figure 2c). In DSEM samples, TfR2 expression was observed at the cytoplasmic level in neoplastic cells (Figure 2d).

**Figure 3:**
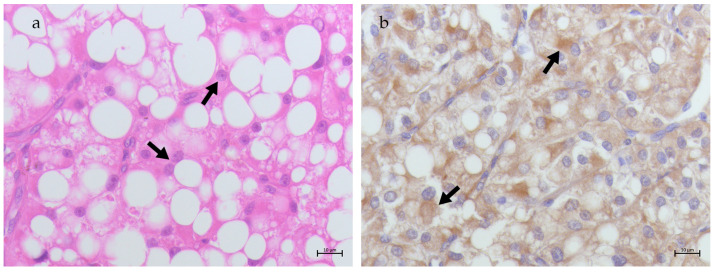
(**a**) Testis, Leydig cell tumor. Neoplastic polygonal cells arranged in cords separated by stroma (fat arrows). H&E 40× bar 10 μm; (**b**) TfR2 immunostaining in LCT: cytoplasmic signal observed in neoplastic Leydig cells (fat arrows); 40× bar 10 μm.

*Leydig cell tumors*—LCT samples (S24–S26) showed polygonal cells with round nuclei and prominent central nucleoli, abundant eosinophilic highly vacuolized cytoplasm, arranged in cords (Figure 3a). In these samples, neoplastic Leydig cells showed cytoplasmic TfR2 expression (Figure 3b).

**Figure 4:**
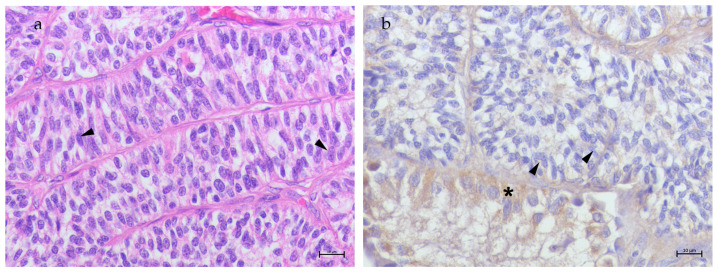
(**a**) Testis, Sertoli cell tumor. Tubules filled only by neoplastic Sertoli cells (arrow heads). H&E 40× bar 10 μm; (**b**) TfR2 immunostaining in SCT: no signal was detected in Sertoli cells within the tubules (arrow heads), while moderate positivity was observed in Sertoli cells of Sertoli-only like tubules (asterisk); 40× bar 10 μm.

*Sertoli Cell Tumors*—SCT samples (S1–S5) were defined by spindle-shaped cells with oval hyperchromatic nuclei and prominent single or multiple nucleoli, while the cytoplasm ranged from eosinophilic to pale. Mitotic figures were rare (Figure 4a). In these samples, no positivity for TfR2 was observed in tumoral cells, while moderate positivity was observed in Sertoli cells of Sertoli-only-like tubules (Figure 4b).

### 3.3. Western Blot Results

**Figure 5:**
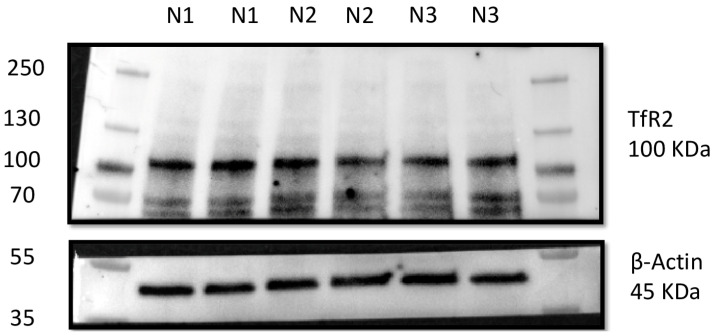
Representative TfR2 immunoblot analysis in non-neoplastic canine testis. Immunoblot analysis performed on the total protein lysates of three non-neoplastic canine testis samples loaded twice. In total, 40 µg of lysates were loaded for each sample, the tag of which is reported on the right of the membrane. The membranes were cut using the protein ladder as a reference and incubated with the primary anti-TfR2 antibody. The immunoreactive band against B-actin was used as a loading control (see also Supplementary Materials).

The specificity of the TfR2 antibody used throughout the study was ensured through Western blotting analysis on the total protein lysates from non-neoplastic canine testis. TfR2-specific antibody gave an immunoreactive band of ~100 KDa in each total protein lysate of the canine testis (Figure 5), showing that this antibody recognizes the canine isoform of TfR2 at the right molecular weight of 100 KDa as previously observed [47,48]. This result demonstrates for the first time the specificity of this antibody in selective immunoreactions with TfR2 in dogs.

## 4. Discussion

Iron metabolism is an essential biological process for maintaining cellular and systemic homeostasis [18]. Iron is a key cofactor in various enzymatic reactions, playing a critical role in oxygen transport [49], energy production [50], and DNA synthesis [51]. In the testis, iron is necessary for spermatogenesis, testosterone production, and the proper functioning of Sertoli and Leydig cells [52]. However, iron could also be a trigger for oxidative stress, in the phenomenon identified as ferroptosis [25]. Iron metabolism dysregulation is increasingly recognized as a hallmark of tumorigenesis [53]. For this reason, iron-related proteins are becoming more and more studied in neoplastic tissues; particularly, great interest is posed on iron uptake proteins such as TfR1 [54,55,56,57]. Less is known about its homolog, TfR2, even though its expression has been reported in various cancer types [46]. In this study, we showed that the expression of TfR2 in canine non-neoplastic testis is limited to Sertoli cells and, to a lesser extent to spermatogonia and spermatozoa, which, interestingly, also express TfR1 [32]. The contemporary expression of TfR1 and Tfr2 in spermatogonia confirms that developing germ cells require high amounts of iron to support their numerous proliferative divisions [26] and to meet this demand they increase iron uptake, preferentially through TfR1 [32]. Also, we reported here for the first time a constant but low cytoplasmatic positivity for TfR2 in Sertoli cells, where TfR1 is also highly expressed [32]. This appears particularly interesting as Transferrin Receptors (TfRs) are generally described as highly active in proliferating cells, while relatively downregulated in mature cells like SCs [58]. Although some early studies have questioned the presence of TfRs in Sertoli cells in both rats and humans [59,60], our data reported in the present and our previous study in dogs agree with what was observed by others [58], underlining the peculiarity of canine SCs and suggesting their role in providing iron to developing germ cells. However, future studies on the role of iron in the complex mechanism of supporting the growth and differentiation of germ cells elicited by Sertoli cells should necessarily involve a broader molecular panel.

In addition to its emerging role in non-neoplastic testicular canine tissues, TfR2 expression in neoplastic testicular tissues revealed intriguing and unexpected patterns; most notably, they suggest that TfR2 was preferentially expressed in germ cell tumors, representing a peculiar feature of this type of neoplasia in dogs. In our previous study, we highlighted that TfR1 expression in ITSEMs was limited to Sertoli-like cells, with no detectable signal in neoplastic germ cells, whereas it was robustly expressed in neoplastic germ cells of DSEMs [32]. Conversely, in the present study, TfR2 was strongly expressed in both ITSEMs and DSEMs cells. Since, to the best of our knowledge, such a behavior is described here for the first time, we have no terms of comparison and can only attempt some speculations keeping in mind the known biological role of TfRs and, in particular, of TfR2. In fact, it is known that TfR2 has a regulatory function on intra and extra-cytoplasmic iron levels, through the modulation of hepcidin [61,62,63,64,65,66,67,68]. A previous study by Wang et al. [69] was the first to demonstrate hepcidin mRNA expression in human testicular tumors, indicating a potential role for hepcidin in the progression of these cancers. Thus, we cannot exclude the atypical activation of the hepcidin molecular circuit in canine SEMs, since it could contribute to maintaining high iron cytoplasmic levels favoring neoplastic growth by preventing the release of iron from the cell to circulating Tf. However, in ITSEMs, the elevated TfR2 expression in the absence of TfR1 may also suggest a compensatory mechanism in response to impaired iron metabolism, potentially driving tumor progression and malignancy through atypical activation of TfR2 pathways. Also, the concurrent expression of TfR1 and TfR2 in neoplastic germ cells of DSEMs could point to an increased demand for iron to sustain a higher proliferation rate [70,71]. Further studies in this direction will help us better understand which molecular pathways related to iron metabolism are actually activated in these canine tumors.

Regarding the LCT and SCT, while LCT showed low positivity for TfR2 compared to normal Leydig cells, intriguing results were observed in SCTs: the absence of TfR2 expression. This aligns with previous findings [32], which reported a lack of expression for other iron-related proteins, including TfR1, FTH1, and occasional NCOA4 expression. Given the critical role of Sertoli cells in mediating iron exchange with germinal cells for normal spermatogenesis [72,73], the absence of germinal cells in SCTs likely disrupts their “nurse cell” function and alters the iron flow. Consequently, Sertoli cells in these tumors appear to reduce the expression of iron uptake proteins which could justify the low proliferation rates characterizing SCTs [70,74]. The lack of TfR2 expression strengthens the evidence for altered iron metabolism in SCTs and highlights the need for comprehensive studies on the iron-protein network in these tumors.

TfR2 proved expression in canine SEMs is an important finding for therapy. Currently, bilateral castration is the treatment of choice for testicular tumors in dogs, while chemotherapy and radiotherapy are still discussed as only limited studies in metastatic or highly aggressive SEMs are available [75,76]. However, collateral effects are often associated with castration such as prostate cancer, lymphosarcoma, and joint diseases [77,78,79]. Therefore, specific delivery of therapeutic molecules via receptor-mediated endocytosis into malignant cells could reduce those systemic effects [54]. While the use of TfR1 has already been established [54], less is known about TfR2 in cancer therapy [80]. Our data support the opinion that TfR2 could specifically target SEMs neoplastic cells given its higher expression in those cancer types. Among the existing antineoplastic drugs, cisplatin has been widely used to treat human testicular cancer [81,82] and canine SEMs [75], being generally well tolerated by most canine patients [83]. Cisplatin can induce ferroptosis, an iron-dependent cell death [84], by disrupting the glutathione-dependent antioxidant mechanisms, leading to uncontrolled lipid peroxidation and subsequently to cell death [85]. Considering the specificity of TfR2 in SEMs and the possibility of conjugating cisplatin to Tf [86], it could be possible to increase selectivity and intracellular drug concentration in these tumors by administering this conjugated to decrease overall toxicity, especially in metastatic and more aggressive forms [54].

## 5. Conclusions

For the first time, this study highlights the expression of TfR2 in canine testicular tissue, revealing its potential relevance, especially in SEMs. Besides suggesting a new possible therapeutic target, our results could open the road to new translational studies in human oncology.

## Figures and Tables

**Table 1:** Breeds, age and diagnosis of 29 canine testis samples.

Samples	Breed	Age (ys)	Histological Diagnosis
S1	Poodle	5	SCT
S2	English Setter	9	SCT
S3	English Setter	9	SCT
S4	Mixed Breed	6	SCT
S5	German Sheperd	7	SCT
S6	Mixed Breed	12	ITSEM
S7	Mixed Breed	10	ITSEM
S8	Pit bull	12	ITSEM
S9	Mixed Breed	11	ITSEM
S10	Mixed Breed	11	ITSEM
S11	Mixed Breed	8	ITSEM
S12	Beagle	11	DSEM
S13	German Sheperd	8	DSEM
S14	German Sheperd	10	DSEM
S15	Mixed Breed	6	DSEM
S16	Mixed Breed	16	DSEM
S17	West Highland Terrier	7	DSEM
S18	Poodle	5	DSEM
S19	English Setter	9	DSEM
S20	English Setter	9	DSEM
S21	Mixed Breed	6	DSEM
S22	German Sheperd	7	DSEM
S23	Mixed Breed	8	DSEM
S24	Mixed Breed	9	LCT
S25	Mixed Breed	8	LCT
S26	German Sheperd	10	LCT
N1	Poodle	8	N.n. testis
N2	German Sheperd	9	N.n. testis
N3	English Setter	7	N.n. testis

SCT: Sertoli cell tumor; ITSEM: Intratubular seminoma; DSEM: Diffuse seminoma; LCT: Leydig cell tumor; N.n.: Non-neoplastic.

**Table 2:** Primary antibody used for immunohistochemistry analysis.

Antibody	Manufacturer/Clone	Host Species	Dilution
TfR2	Antibodies */Polyclonal	Rabbit	1:100

* https://www.antibodies-online.com/antibody/2782221/anti-Transferrin+Receptor+2+TFR2+N-Term+antibody/ (accessed 12 November 2024).

**Table 3:** Primary antibody used for Western blot analysis.

Antibody	Manufacturer/Clone	Host Species	Dilution
TfR2	Antibodies */Polyclonal	Rabbit	1:1000

* https://www.antibodies-online.com/antibody/2782221/anti-Transferrin+Receptor+2+TFR2+N-Term+antibody/ (accessed 12 November 2024).

## Data Availability

Further information on the data included in this study is available from the corresponding author upon reasonable request.

## Supplementary Materials

The following supporting information can be downloaded at: https://www.mdpi.com/article/10.3390/ani15020264/s1, Figure S1: WB image.
