# Supplementary material for: Transferrin Receptor 2 in Canine Testicular Tumors: An Emerging Key Role in Seminomas
Source: Animals (Basel). 2025 Jan 18;15(2):264. doi: 10.3390/ani15020264 (PMC11758335; doi:10.3390/ani15020264)
Supplement: Supplementary file 1 [file animals-15-00264-s001.zip › animals-3423739-supplementary.pdf]

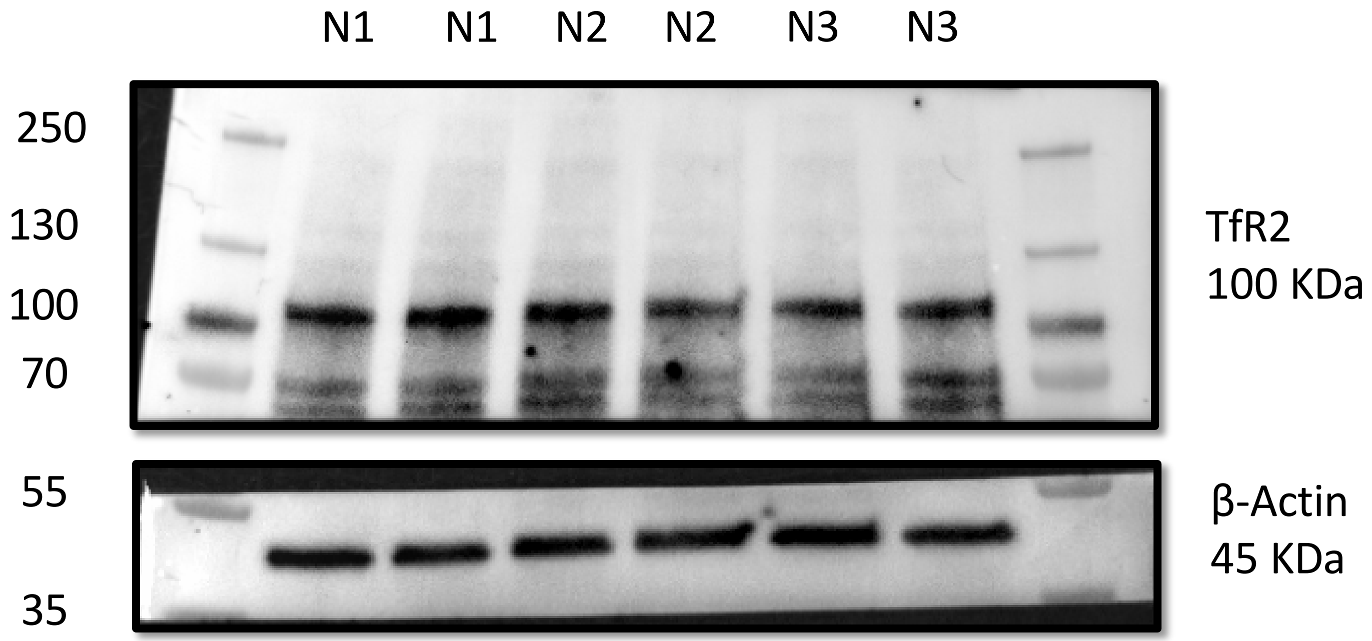

Figure S1. Rapresentative immunoblot analysis of TfR2 and β-Actin on total protein lysates of three non-neoplastic canine testis (N1-N3), each one loaded twice. The full membrane of the Western blot was cut at approximately 70 kDa to allow the testing of both antibodies on the same membrane, as the two target proteins have different molecular weights (100 kDa for TfR2 and 45 kDa for β-Actin.
